# Embracing Artificial Intelligence (AI) in Nursing Education through Wearable Technology: Innovation-Driven Teaching

**DOI:** 10.17533/udea.iee.v43n2e01

**Published:** 2025-07-15

**Authors:** Agostinho A. C. Araújo, Lucas Gardim, Andrea Bernardes, Isabel Amélia Costa Mendes, Kristina Mikkonen

**Affiliations:** 1 Bachelor of Science in Nursing, Master of Science, PhD Student. Email: agostinhoaraujo@usp.br. Corresponding author. https://orcid.org/0000-0003-0996-0385 Universidade de São Paulo Brazil agostinhoaraujo@usp.br; 2 Registered Nurse, M.Sc Student. Email: lucasgardim@usp.br. https://orcid.org/0000-0002-5949-1663 Universidade de São Paulo Brazil lucasgardim@usp.br; 3 Registered Nurse, Ph.D. Associate Professor. Email: andreab@eerp.usp.br. https://orcid.org/0000-0002-9861-2050 Universidade de São Paulo Brazil andreab@eerp.usp.br; 4 Registered Nurse, Ph.D. Emeritus Professor. Email: iamendes@usp.br. https://orcid.org/0000-0002-0704-4319 Universidade de São Paulo Brazil iamendes@usp.br; 5 Registered Nurse, Ph.D. Professor. Email: https://orcid.org/0000-0002-4355-3428kristina.mikkonen@oulu.fi. University of Oulu Univeristy of Oulu https://orcid.org/0000-0002-4355-3428kristina.mikkonen@oulu.fi; 6 Ribeirão Preto College of Nursing, University of São Paulo, Ribeirão Preto, Brazil. Universidade de São Paulo Ribeirão Preto College of Nursing University of São Paulo Ribeirão Preto Brazil; 7 Research Unit of Health Sciences and Technology, Faculty of Medicine, University of Oulu, Oulu, Finland. University of Oulu Research Unit of Health Sciences and Technology Faculty of Medicine University of Oulu Oulu University of Oulu; 8 Faculty of Nursing, University of Alberta, Edmonton, Canada. University of Alberta Faculty of Nursing University of Alberta Edmonton Canada

## Embracing Artificial Intelligence (AI) in Nursing Education through Wearable Technology: Innovation-Driven Teaching

In the era of technology, forward-thinking educators are critical in advocating for curriculum changes to advance nursing education. As the world becomes increasingly technological, educators must rethink their roles as change agents to meet the evolving needs of contemporary learners. Artificial Intelligence (AI) has emerged as a disruption in educational environments, yet a significant gap remains in scientific evidence regarding the most effective strategies for integrating AI into nursing education. Still, there are a number of challenges faced by students, educators, and universities in using AI. As digital natives, students are continuously exposed to novel technologies but are not adequately guided on the ethical and responsible use of AI. Meanwhile, educators, who are not digital natives, must continually develop their own competencies through specialized training to prepare the next generation of nurses. Simultaneously, universities often lack institutional policies to support the structured incorporation of AI, hindering improvements in learning outcomes. In all instances, resistance to innovation can further delay transformation. As an educational tool built on prompts and language models, AI requires complementary technologies for integration into any context, including nursing education. With the anticipated technological future of healthcare systems, it is essential to explore how AI can be integrated into nursing education through emerging technologies to better prepare the future workforce, with wearables representing a key pathway for leveraging AI.

As technology advances, AI will continue to shape nursing education. However, educators are still unfamiliar with AI, showing a limited understanding of its tools and capabilities.^1^ To understand, interact, and translate AI into education, nurse educators should develop AI literacy. When equipped with the competencies required, they can choose the intelligent systems that best meet students' needs and preferences, establish clear guidelines, and prepare students to navigate ethical dilemmas in technology-driven health systems. It can support the development of nursing students' professional identity values, maximizing students’ retention. Since professional identity is a critical factor that influences nursing student retention,^2^ fostering positive faculty-student relationships becomes essential for shaping students' professional self-concept and commitment.^3^^-^^4^ They can also leverage AI to bridge the theory-practice gap, fostering an educational environment where students feel empowered to engage deeply and adapt skillfully to evolving challenges.^5^^-^^7^ By providing personalized learning experiences,^8^^-^^9^ AI in nursing education is well positioned to enhance learning outcomes,^5^^,^^10^ leading to improved student's satisfaction and academic performance.^11^ Yet, its integration raises significant ethical and privacy concerns that require careful consideration by academic decision-makers. A central concern is the ethical management of sensitive data, as nursing education often involves using personal and patient-related information, requiring strict adherence to privacy standards. Furthermore, biased AI algorithms reinforce existing inequalities in education, making it challenging to ensure equitable learning opportunities. Addressing these issues requires institutions to incorporate ethical principles, such as autonomy, nonmaleficence, beneficence, justice, and explainability into the curriculum, which not only ensures compliance with cyber ethics but also promotes the responsible use of AI.^8^^,^^12^ While AI promises to transform various aspects of educational, academic, and clinical environments, many educators remain unaware of the benefits of other emerging technologies also shaping nursing education, such as wearable technology.

Wearables are electronic devices designed to be worn as accessories, including smart glasses, smartwatches, and smart clothing. These devices offer a range of functionalities, with health monitoring being particularly notable. They can track sleep quality, monitor physical activities, provide protection, initiate actions (calling emergency services in dangerous situations, for instance), and assist in managing personal tasks.^13^ In nursing education, wearable devices provide real-time monitoring, simulation of patient scenarios, and hands-on training for students to develop skills in a safe and controlled environment.^14^ Integrating wearable technology into nursing education requires alignment with student needs and nursing curriculum outcomes. When combined with other realist pedagogical approaches (e.g., simulation labs), wearable technology promises a transformation in how students engage with their learning environments.^14^ Wearables offer an immersive, hands-on approach that aligns with the learning preferences of digital-native students who expect interactive technology-driven experiences.^14^^-^^15^ This alignment is critical, as it suggests that wearable technology in education is not merely a novel addition but a response to shifting expectations and cognitive habits shaped by a digitally integrated world. By embedding wearables in the educational experience, students are encouraged to actively participate, apply knowledge in real-time contexts, and develop professional skills with a nuanced understanding of their applicability in clinical environments. Although these devices cannot be incorporated into supervised practices or internships in a hospital environment due to the risk of contamination,^16^ they can be incorporated into simulated scenarios, providing a practical educational strategy to enhance nursing students' competencies before their immersion in the clinical setting.^14^ Simulated scenarios in nursing education are a valuable approach to developing competencies,^17^ which can be further enhanced through the use of wearables.^14^ Wearables can serve as a bridge between theory and practice, fostering personalized and immersive learning experiences, and ultimately pivoting nursing education: driving change in educational strategy while maintaining the foundations of nursing education.

Education must continuously evolve by adopting new pedagogical approaches and incorporating AI-driven tools.^18^ Embracing AI in nursing education through wearable technology does not go against nursing education's core values. Instead, it enables students’ new forms of knowledge acquisition, bridging the gap between theory and practice in nursing education. AI-driven wearables can optimize teaching resources and enhance human interaction, representing an emerging strategy for leveraging AI. Although AI represents a key pathway forward in nursing education, there are considerable barriers rooted in cultural resistance to innovative teaching methods. While challenges are still faced by students, educators and universities in integrating AI in nursing education, decision-makers can contribute to the establishment of institutional policies that can strengthen the curriculum to prepare nursing students to meet the demands of technology-driven healthcare systems, where technology and patient care are increasingly intertwined. With a significant gap in scientific evidence regarding the most effective strategies for integrating AI into nursing education, further research is required to explore the role of wearable technology in developing competencies among nursing students, ensuring a comprehensive understanding of its impact on educational outcomes.
